# Case report: atypical fungal obstruction of the left ventricular assist device outflow cannula

**DOI:** 10.1186/1749-8090-9-40

**Published:** 2014-02-25

**Authors:** Jiri Maly, Ondrej Szarszoi, Zora Dorazilova, Josef Besik, Martin Pokorny, Tomas Kotulak, Ivan Netuka

**Affiliations:** 1 Department of Cardiovascular Surgery, Institute for Clinical and Experimental Medicine, Vídeňská 1958/9 140 21 Prague 4, Prague, Czech Republic; 2 Department of Anesthesiology and Intensive Care Medicine, Institute for Clinical and Experimental Medicine, Prague, Czech Republic

**Keywords:** Left ventricular assist device, Outflow cannula obstruction, Fungal infection, Thrombus formation

## Abstract

We describe a very rare case of outflow cannula obstruction with fungal infectious thrombus formation. Discussion includes the etiology, diagnosis, and management of fungal infection complications related with long-term mechanical circulatory support. Left ventricular assist devices (LVADs) are increasingly used as bridge to transplant and permanent long-term therapy in the population with end-stage heart failure. Even though better clinical outcomes have been achieved with the newer-generation continuous-flow devices, infection complications are still a major risk for patients with continuous-flow LVAD implantation in long-term follow-up [Ann Thorac Surg 90:1270-1277, 2010]. Device-related infections can be categorized as driveline infections, pump-pocket infections, and LVAD-associated endocarditis [Expert Rev Med Devices 8: 627-634, 2011]. The microbiological profile is very heterogeneous; the most common pathogens are Staphylococcus, Pseudomonas, Streptococcus species, and Candida. Severe fungal infection may lead to dysfunction of the LVAD due to obstructive mass formation within the device. Due to the only anecdotal reports in the current literature, we present a very rare case of outflow fungal infectious thrombus formation leading to outflow cannula obstruction in patient with LVAD.

## Background

Left ventricular assist devices (LVADs) are increasingly used as bridge to transplant and permanent long-term therapy in the population with end-stage heart failure. Even though better clinical outcomes have been achieved with the newer-generation continuous-flow devices, infection complications are still a major risk for patients with continuous-flow LVAD implantation in long-term follow-up [1]. Device-related infections can be categorized as driveline infections, pump-pocket infections, and LVAD-associated endocarditis [2]. The microbiological profile is very heterogeneous; the most common pathogens are Staphylococcus, Pseudomonas, Streptococcus species, and Candida [1,2]. Severe fungal infection may lead to dysfunction of the LVAD due to obstructive mass formation within the device.

## Case presentation

A 56-year-old caucasian male with dilated cardiomyopathy was previously treated with an implantable cardiac defibrillator (ICD) as secondary prevention therapy (2002) and with a combined system of cardiac resynchronization therapy (CRT) and ICD—CRT/ICD therapy (2009) with several infectious complications, which led to explantation of the system and reimplantation of the CRT/ICD (surgically implanted epicardial electrode; 2010).

The patient was admitted to our institution (12/2010) from a referral hospital in cardiogenic shock with arrhythmic storm triggered with incessant monomorphic ventricular arrhythmia. Radiofrequency (RF) ablation was unsuccessful, so urgent implantation of an LVAD—HeartMate II (Thoratec Corporation, Pleasanton, CA, USA) was indicated. The procedure was performed in a standard fashion under transesophageal echocardiogram (TEE) guidance together with De Vega tricuspid valve annuloplasty and cryodestruction of arrhythmogenic substrate.

During the entire postoperative period, repeated life-threatening septic complications were observed. The patient was treated gradually with antibiotics to 02/2011 (meropenem, linezolid, fluconazol, anidulafunginum, cefepime, ampiciline, micafungin, piperacilline/tazobactam). Computed tomography (CT) scans showed signs of pulmonary interstitial pneumonia in both lungs from 12/2010 to 2/2011. During this period, it was necessary to intubate the patient several times because of respiratory insufficiency. Clinical signs of infection disappeared after prolonged treatment with broad-spectrum antibiotics. In early 2/2011 CT scans, bronchoscopy with lavage, and blood cultures were negative.

The patient also had repeated gastrointestinal bleeding that required blood transfusion. Colonoscopy showed no clear source of bleeding—inflammation and small surface ulceration of the colon, two small sores in rectal ampulla, and normal gastric fibroscopy. Parenteral nutrition and intermittent nasogastric tube were used.

Following the gradual rehabilitation and education of the patient about operating the LVAD equipment and dressing the driveline exit site, he was discharged to home. Calculated LVAD flow ranged between 5.0 and 6.0 L/min. During the entire duration of circulatory support, no LVAD suction events were detected, pump power consumption remained in the normal range (6 to 7.5 watts at pump speed 9,400 rpm), and the patient was listed for heart transplantation. We did not observe any signs of hemolysis; anticoagulation levels remained stable, and the bilirubin levels throughout postoperative follow-up were within normal range. The patient had no signs of active systemic infection.

**Figure 1:**
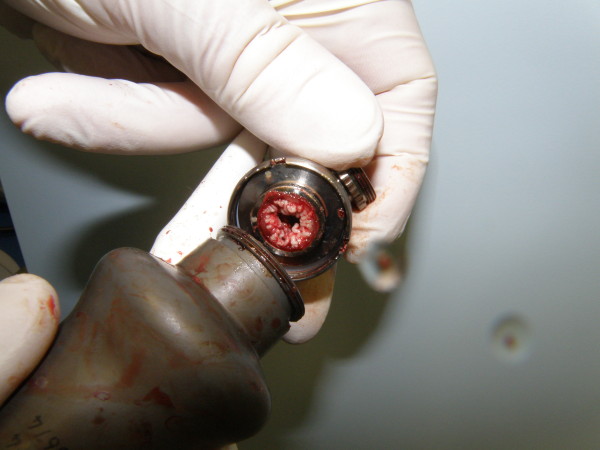
Explanted LVAD during heart transplant.

**Figure 2:**
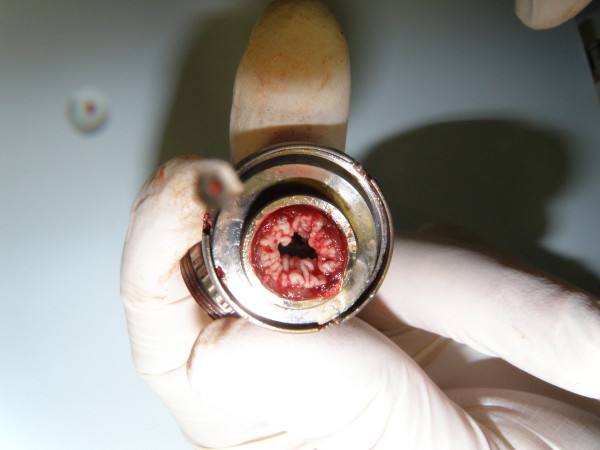
Detail of the infectious thrombus formation in the LVAD.

**Figure 3:**
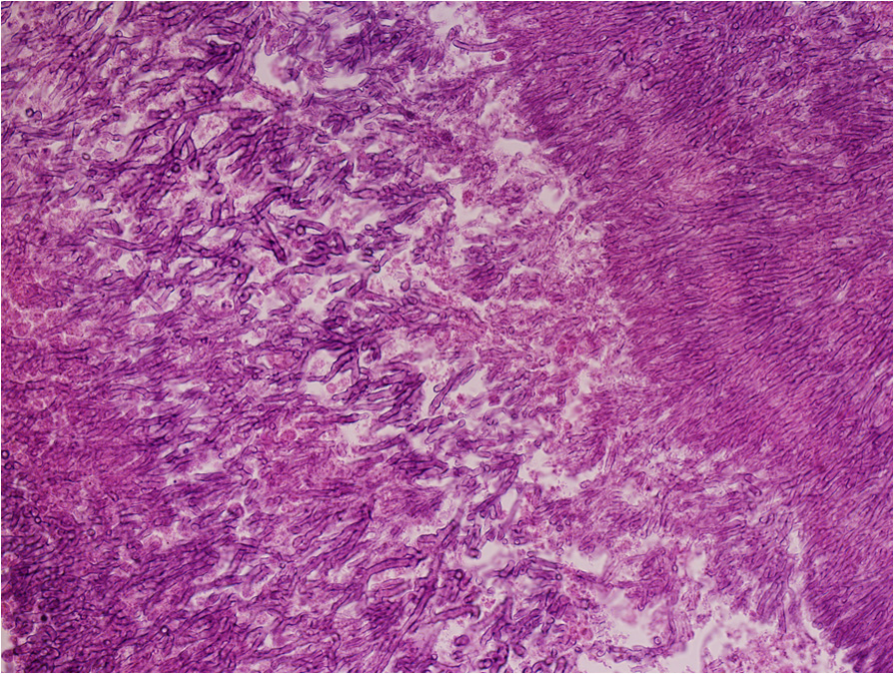
Microscopic sample of the thrombus shoving aspergilus hyphae.

The patient was rehospitalized in 3/2011 due to worsening of his condition. He was afebrile, fatigued, had a dry cough and brown discoloration of urine. Hemoglobinuria, an increase of inflammatory markers, and mild alteration in hepatic and renal function were detected. Initial blood cultures were negative. Transthoracic echocardiogram (TTE) and TEE imaging showed no obstruction of the inflow or outflow cannulas, but there was a decrease in flow velocities (inflow velocity = 35 cm/s; outflow velocity = 70 cm/s). The left ventricle was severely dilated and hypokinetic. The aortic valve was opening at 1:1. Even at high-speed operation (11,600 rpm), the left ventricle could not be unloaded with closure of the aortic valve. Systemic thrombolysis was considered, but a donor heart became available (patient was listed as an urgent candidate) and heart transplantation was performed in 4/2011. Examination of the explanted LVAD revealed thrombotic-like obstruction of the outflow cannula (Figures 1 and 2). Histopathologic examination and microbiological culturing of the mass have showed extensive fungal growth (Figure 3). The thrombus showed fibrinous debris, scattered inflammatory cells, and hyphae with the presence of Aspergillus species.

The patient was discharged from the hospital eight weeks after the transplantation. During the first 12 months, patient was repeatedly hospitalized due to soft tissue wound infections. Treatment consisted of soft tissue debridement and V.A.C.^®^ Therapy. *Candida albicans* was culture-confirmed in multiple samples. Therefore, the patient was aggressively treated with antifungal therapy in the first three months with intrakonazol and then treated for the next nine months with fluconazol. Lower-dose corticosteroid protocol with 5 mg of prednisone daily was administered. After one year, conversion from sirolimus to everolimus was made because of early signs of coronary artery disease in the donor heart. During the two years follow-up, the patient was monitored according to our institutional protocol with regular cardiac biopsies and echocardiogram examinations, without any signs of serious complications.

## Discussion and conclusions

With increasing durations of LVAD support, the accurate diagnosis of device malfunction is crucial [2]. Diagnosis of fungemia is often difficult, and blood cultures are negative in about half of the samples from patients with active infection. To find the source of a fungal infection and select the most appropriate treatment is often challenging. Fungal pathogens, most frequently Candida species, can cause LVAD-associated endocarditis [2]. Unfortunately, the only clinical sign of a thrombotic fungal infection is often fever or sepsis of unknown origin. TTE and TEE imaging are commonly used to follow up LVAD patients [3]. Currently, the suggested normal range for outflow graft velocities is 1.0–2.0 m/s. Abnormal values can eventually identify device malfunction along with overall evaluation of LVAD function, such as dilated left ventricle, rightward septal shift, mitral regurgitation, or ejection through aortic valve. In summary, pulsatile flow through the aortic valve, low-velocity antegrade flow in outflow cannula on TTE or TEE images, and thrombotic material detected on CT scan can refine the diagnosis [4]. We can also evaluate data from the HeartMate II controller, such as speed, power, pulsatility index, and flow, which relate to device function. In order to reveal graft obstruction, there was designed a complex examination pattern, where the majority of previously mentioned variables are incorporated - RAMP test [5]. However, borderline echocardiographic findings together with clinical status are not specific enough to identify outflow graft fungal occlusion [4].

According to the recent guidelines, in our institution, we administer fluconazol as a *Candida* infection prophylaxis to all LVAD paints, during high-risk periods. Aspergillosis prophylaxis is not routinely recommended. In case of proved fungal infection the early initiation of effective should be critical. Patients afflicted by *Candida* endocarditis should be immediately treated with echinocandin. In patients with suspected invasive aspergillosis voriconazole is administered as the first choice therapy [6]. Here should be emphasized that eradication of persistent fungemia in patient with implanted LVAD is nearly impossible without its removal.

In our case, the major pathogen was Aspergillus species, which was identified through histopathological methods. Clinical manifestation of Aspergillus infection is uncommon. When it does occur, it is almost always in the setting with an immunocompromised patient. Filamentous fungi as a pathogen are infrequently associated with device infection. In earlier presented case series of Aspergillus infections in LVAD patients, there was clinical evidence of outflow-tract obstruction related to overwhelming device infection similar to our case. Current imaging techniques, either CT scan or echocardiography, were limited in their ability to exactly detect LVAD-associated endocarditis in our patient. Definitive diagnosis was established from species identification of the obstructive mass in the internal surface of the outflow cannula following explantation of the device. It should be stressed that tissue specimens from the device must always undergo histopathological examination.

Current data are limited, but it is confirmed that combined medical and surgical therapy generally should be the preferred treatment for fungal endocarditis. Although there are no currently available evidence-based guidelines regarding LVAD endocarditis, we believe that clinical decision-making in patients with signs of ongoing sepsis and end-organ dysfunction together with any signs of LVAD malfunction should be very straightforward in terms of device exchange or explantation. In patients with VADs and severe fungal infection who cannot be explanted electively, an urgent transplant listing plays key role. Due to organ donors shortage VAD exchange via left subcostal incision is increasingly used.

## Consent

Written informed consent was obtained from the patient for publication of this case report and any accompanying images. A copy of the written consent is available for review by the Editor-in-Chief of this journal.

## Abbreviations

LVAD: Left ventricular assist device; ICD: Implantable cardiac defibrillator; CRT: Cardiac resynchronization therapy; RF: Radio-frequency; TEE: Transesophageal echocardiogram; TTE: Transthoracic echocardiogram; CT: Computer tomography.

## Competing interests

The authors whose names are listed immediately below certify that they have no affiliations with or involvement in any organization or entity with any financial interest, or non-financial interest in the subject matter or materials discussed in this manuscript. Authors: Jiri Maly, MD, PhD; Ondrej Szarszoi, MD, PhD; Zora Dorazilova, MD; Josef Besik MD, PhD; Martin Pokorny, MD; Tomas Kotulak MD; Ivan Netuka MD, PhD.

## Authors’ contributions

JM Operated on the patient, main author. OS Operated on the patient, co-author. ZD the patient was followed-up in her cardiological ambulance after LVAD implantation, co-author. JB the patient was followed-up in his surgical ambulance after LVAD implantation, co-author. MP took care of the patient on ICU, co-author and drafted the manuscript. TK Anaesthetist, co-author. IN Operated on the patient, co-author. All authors read and approved the final manuscript.
